# Ethical, legal and social/societal implications (ELSI) of recall-by-genotype (RbG) and genotype-driven-research (GDR) approaches: a scoping review

**DOI:** 10.1038/s41431-022-01120-y

**Published:** 2022-06-15

**Authors:** Katharina Tschigg, Luca Consoli, Roberta Biasiotto, Deborah Mascalzoni

**Affiliations:** 1 Department of Cellular, Computational, and Integrative Biology, University of Trento, Trento, Italy; 2 Institute for Biomedicine & Affiliated Institute of the University of Lübeck, Eurac Research, Bolzano, Italy, Bozen, Italy; 3 Institute for Science in Society, Radboud University, Nijmegen, Netherlands; 4 Department of Biomedical, Metabolic and Neural Sciences, University of Modena and Reggio Emilia, Modena, Italy; 5 Department of Public Health and Caring Sciences, Center for Research Ethics and Bioethics, Uppsala University, Uppsala, Sweden

**Keywords:** Ethics, Medical genomics

## Abstract

Recall by Genotype (RbG), Genotype-driven-recall (GDR), and Genotype-based-recall (GBR) strategies are increasingly used to conduct genomic or biobanking sub-studies that single out participants as eligible because of their specific individual genotypic information. However, existing regulatory and governance frameworks do not apply to all aspects of genotype-driven research approaches. The recall strategies disclose or withhold personal genotypic information with uncertain clinical utility. Accordingly, this scoping review aims to identify peculiar, explicit and implicit ethical, legal, and societal/social implications (ELSI) of RbG study designs. We conducted a systematic literature search of three electronic databases from November 2020 to February 2021. We investigated qualitative and quantitative research methods used to report ELSI aspects in RbG research. Congruent with other research findings, we identified a lack of qualitative research investigating the particular ELSI challenges with RbG. We included and analysed the content of twenty-five publications. We found a consensus on RbG posing significant ethical issues, dilemmas, barriers, concerns and societal challenges. However, we found that the approaches to disclosure and study-specific recall and communication strategies employed consent models and Return of Research Results (RoRR) policies varied considerably. Furthermore, we identified a high heterogeneity in perspectives of participants and experts about ELSI of study-specific RbG policies. Therefore, further fine-mapping through qualitative and empirical research is needed to draw conclusions and re-fine ELSI frameworks.

## Background

For more than 15 years, Next-Generation Sequencing (NGS), Whole-Genome-Sequencing (WGS) and Genome-Wide-Association- Studies (GWAS) powered the integration of genomic data, enabled personalised medicine approaches, and led to an increase of scientific knowledge translation from biology to the societal dimension (communicating genetic risk to the individual participant) [1–3]. To further analyse the vast amounts of genotypic data, targeted bottom-up approaches to select participants are gaining popularity versus conventional random sampling strategies [4–7]. Recall by Genotype (RbG), and Genotype-driven-research (GDR) strategies are bottom-up models to recall participants for genomic research and phenotyping selectively based on the presence or absence of a specific genotypic variant. Genotype-driven selection strategies pose a powerful tool for identifying causalities between genes and diseases, specifically in cases when genotypes are rare, and phenotyping of extensive sampling frames would be too costly [6, 8, 9]. Furthermore, when studying human subjects with specific genotypes, there is a higher probability of detecting underlying disease mechanisms and genetic associations, even though defining risks for the individuals among the identified variants is not easy [7, 8].

Similarly, defining study policies respective to the recruitment/recall phase, the consent procedures, and the Return of Research Results (RoRR) policies is challenging as RbG approaches have not been outlined fully and are bound to the consent and context of the original or parent study [5, 10]. The RbG study design relies on dividing participants of an original large scale study into smaller groups; accordingly, a crucial balance between sample size and statistical power must be kept [1]. Moreover, the economic benefit of decreasing sample sizes for RbG is weighed against the particular ELSI considerations that arise, including the risks emerging from classification practices in genomics, biased datasets and a lack of diversity, and the risk of genetic discrimination and stigmatisation [1, 8, 11–15]. There is a need to have further discussions on the ethical issues involved in RbG [16].

We conducted a scoping review to identify ethical issues and debates with nuanced ELSI considerations regarding different frameworks’ scientific and societal utility to guide the complexities of RbG study design decisions. Considering that ethics goes beyond complying with current legal and regulatory requirements, we will discuss uncertainty, unaddressed issues, diverging study design considerations and missing recommendations of ELSI of RbG.

### Methodology and objective

We used the scoping review methodology [17] and reported it according to PRISMA guidelines [18]. According to PRISMA guidelines, we conducted the search from November 2020 to February 2021.

### Identifying the research question

The main objective of this scoping review is to identify peculiar, explicit and implicit ethical, legal, and societal/social implications (ELSI) of RbG study designs and then discuss the following review questions:

1. What are ethical, legal, societal or social (ELSI) aspects of RbG research?
  - How do different approaches to RbG studies handle the identified ELSI issues in terms of disclosure strategy, study-specific recall and communication strategies, employed consent models and Return of Research Results (RoRR) policies?
2. What type of qualitative and quantitative research and methods were used to report ELSI aspects in RbG research?
3. What are the ELSI debates, issues and future concerns collected from participants and other stakeholders in RbG research?

To synthesise these review questions, we will discuss consensus, conflicts and diverging recommendations on ELSI of RbG that need further investigation.

### Search strategy

**Fig. 1:**
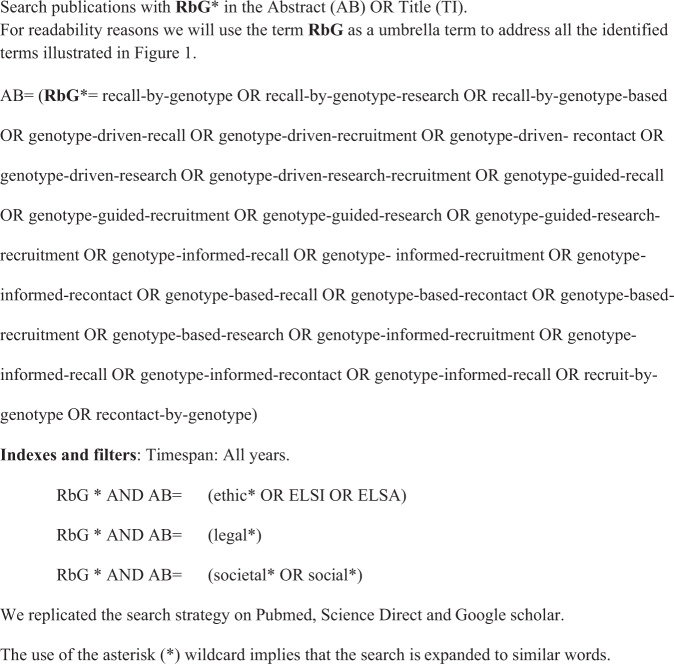
Detailed search strategy (Web of Science) and search terms and strings. Search strategy and search terms.

As a prerequisite to the scoping review, we identified all the relevant terms for RbG studies through iterative search runs in the different databases, as shown in Fig. 1. The databases searched were Web of Science, PubMed (Medical Literature Analysis and Retrieval System Online, MEDLINE), Science Direct, and Google Scholar. The search included all types of documents.

### Article selection and eligibility criteria

**Fig. 2:**
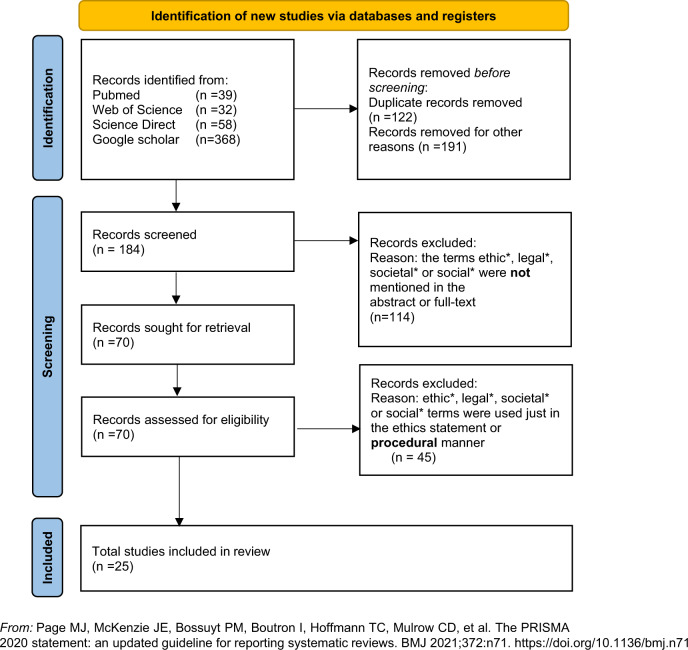
PRISMA 2020 flow diagram for updated systematic reviews which included searches of databases and registers only. Identification of relevant literature. PRISMA referred flowchart about the process of searching and identifying relevant literature. *The use of the asterisk (*) wildcard implies that the search is expanded to similar words.

We included documents written in English. We excluded publications with a clinical focus that did not discuss ELSI aspects (for example, publications that did not discuss ELSI aspects beyond the explicit reporting of compliance and procedural ethical approval processes). We included publications with qualitative and quantitative methods and empirical elements. Exclusions were confirmed by using Endnote X9 and Rayyan filters and manual review. We did not apply any time limitations. KT performed the search and screening. The co-authors verified the screening for accuracy. The selection of eligible and relevant literature was discussed among the authors. Seventy publications were selected for full-text screening and assessed for eligibility. We included 25 publications in the synthesis of the review. Figure 2 demonstrates the detailed selection process and the eligibility criteria formulated to identify relevant publications that address ELSI in RbG.

### Data extraction, charting and synthesis

Publications were retrieved, organised and managed with Endnote X9 and Rayyan to track eligibility decisions systematically [19, 20]. We charted the eligible selection into Microsoft Excel and Word tables to extract data. KT conducted the content analysis with an inductive approach to analyse the contextual use of the searched terms and compile a matrix of themes and considerations from the publications. The identified themes were discussed among the authors and agreed upon.

## Results

Following a full-text review, we included 25 studies in the synthesis (as shown in Appendix) [4–10, 19–36]. We identified an overall lack of qualitative research investigating the ELSI of RbG. There is a consensus from the literature that RbG poses significant ethical and societal challenges with ELSI discussions centred on the themes shown in Table 1. Out of the 25 included publications, we discovered nine publications with empirical data collection methods on ELSI of RbG, as shown in Table 2. We ordered the tables chronologically to demonstrate the evolution of the different studies, some of which were in direct response to another.

**Table 1:** Main themes identified with the content and thematic analysis in the included publications (*n* = 25). This is a map of the results. The table shows the themes and the specific sub-themes found in the literature described with the type of article and the method.

Main themes in RbG research	Themes addressed by the publications		Results
	Description of identified ELSI discussion on	Specific consideration or recommendation	
Study design, stakeholder engagement, and policy development	Ethical balance	Between scientific interests and the participant's rights and preferences	[5, 7, 10, 20, 21, 25, 26, 28, 32, 35]
		Protecting research participants while avoiding overly restrictive policies	
		Balance the potential harms with or without disclosure	
	Scientific and statistical considerations	About RbG compared to other recruitment frameworks	[4]
	ELSI considerations for the study design and how to tailor approaches to the context and cohort	How to translate ELSI considerations into practical RbG policies	[28, 31]
		Practicalities of incorporating genotypic data into population-based study	[5]
		Ethical implications of familial research in RbG	[21]
		Implications of bottom-up approach	[22]
		Practicalities of linking genotype information with electronic health record (EHR)	[9, 10, 36]
Recall frameworks: invitation of participants	Explicit and implicit disclosure by invitation: ethical principle of autonomy pitched against the right not to know unwanted genetic information	How to avoid deception and inform participants (about study purpose, eligibility criteria and the return of research results policy)	[7, 10, 23, 25, 27, 28, 31, 33–35]
		Practicalities of no-disclosure of the targeted genetic variant	[10, 23, 33–35]
Consent procedures	Informed consent [1, 3–5, 7, 21, 22]	Harmful or deceptive characteristic if research is conducted in the absence of disclosure and informed consent	[10, 34]
		Dynamic consent	[31]
		“Presumed” consent	[6]
Return of research results policies (RoRR)	How to return unsubstantiated, uncertain, unexpected, incidental or indeterminate findings and research results [1–5, 8, 10, 16, 17, 22, 24, 25]	How to communicate the details of research results (personal and clinical utility)	[7, 10, 19, 20, 24–26, 28, 29, 32–34]
		“No return of results” policy	
Risks and uncertainties	Potential distress triggered by the invitation or study participation	Disclosure of eligibility criterions can lead to differently derived meaning in patients and participants	[5, 19, 23–25, 29]
		Communication about distinct risks	[21]
		ELSI issues linked to benefits and risks associated with sharing genomic data	[30]
		Uncertainty about how genetic information will be used in the future	
		Discrimination	
Introduce new techniques to society	Whether ethics-related recommendations suffice for broader use of RbG approaches		[31]
	Pediatric RbG		[20]
	Development of tools	To promote education, dissemination and public engagement	[31]
		Less intrusive but faster and more efficient recruitment through electronic tools	
	Careful societal considerations about specific populations		[8]
	Electronic health record and other information are needed for artificial intelligence to integrate genetic and non-genetic information		

Most publications focused ELSI concerns on the Recruitment phase, couples with the RoRR policies because research results from an original study are the basis for identifying and recalling participants for further investigation with the specific RbG study. Another recurring discussion regarding the ethical duty to disclose results (and which types of results) and the ethical issues and concerns regarding explicit and implicit disclosure of research results as carrier status came up. Further, publications reported ethical barriers and challenges concerning the different suitable consent procedures or the lack thereof. The theme of how to tailor procedures to the context of the specific study was discussed by empirical studies searching for balances and using anticipatory research to adjust the study design and policies to the context.

### RbG research and corresponding methods used to explore ELSI aspects

Since 2002 there has been an increase in the use of RbG sub-nested in various large-scale genomic research settings. However, except for a review on ethical implications of familial genetic research and RbG, before 2008, there is no consideration of the specific ELSI of RbG [21]. Most identified publications employing and reporting empirical data collection methods are situated in the UK- or US-based research context.

We identified an overall lack of data on the experiences and opinions of various stakeholders such as participants. The employed methodologies to collect stakeholders’ perspectives on ELSI of RbG were qualitative interviews with participants that were either recalled for genotype-driven research or not eligible for the RbG study but purposively sampled [7, 19, 20, 25, 26, 29, 30]. Other literature on stakeholders and experts like researchers, clinicians, policymakers [7, 19, 20, 25, 26] may lack diversity in reporting the perspectives of different stakeholders because all the publications are derived from the UK- or US-based research context. There is a minimal body of research discussing the results of mixed-methods approaches with experts such as members of the Institutional Review Board (IRB) in the US and other well-trained researchers or scholars on RbG [23, 27, 28]. Current ELSI studies about RbG tend to involve stakeholders with a narrow range of characteristics in terms of education and cultural background, as reported in detail in Table 2.

**Table 2:** List and details of the publications employing qualitative and empirical data collection methods to investigate ELSI of RbG (*n* = 9).

Ref	Title of the publications	Year	Method	Recruitment strategy	Sample size	Participation rate	Characteristics of the sample	Country where the study was conducted
[23]	Ethical challenges in genotype-driven research recruitment	2010	Commentary and case presentation of a quantitative RbG study	*The invitation letter was sent to all study participants (n = 975)*.		The quantitative study reported a response from 51 (5.3%). Of these: *37 (72.5%) opted out of any further contact about the follow-up study and 12 (23.5%) called to volunteer for the follow-up study. Two (3.9%) withdrew from the parent study*.		US
[25]	Research participants' perspectives on genotype-driven research recruitment	2011	qualitative study, interviews	Purposive sampling strategy where *approx. half of the included participants had been recontacted for RbG, and the other half was not*.	*n* = 78		As reported, approximately *two-thirds were female, and most were white, non-Hispanic, and college educated (as a function of the sample in the original study)*.	US
[26]	The meaning of genetic research results: reflections from individuals with and without a known genetic disorder	2011	qualitative research, in-depth interviews	*Individuals were selected based on the presence of genetic traits*.	*n* = 24 *(Cystic Fibrosis participants n = 9; Biobank Participants n = 15)*		As reported, all participants in the interview study were *White and non-Hispanic, reflecting the racial and ethnic composition of the study population*. Overall, the respondents were *well educated, particularly the biobank participants*. As reported, several of the biobank participants are themselves *scientific researchers or physicians or had previously worked in scientific research positions*.	US
[19]	Epilepsy patient-participants and genetic research results as "answers"	2011	qualitative research, semi-structured in-depth interviews, part of a multi-site study [1]	Purposive sampling: *about one third of epilepsy patient-participants that had been recontacted about the genotype-driven follow-up study*.	*n* = 29	*Of 26 epilepsy patient-participants eligible for the genotype-driven follow-up study, 9 completed and interview and of 24 patient-participants who were not eligible 20 completed an interview*.	As reported, most of our interviewees were *female, white, non-Hispanic, and college educated*.	US
[20]	Parent perspectives on pediatric genetic research and implications for genotype-driven research recruitment	2011	qualitative research, interviews with parents of epilepsy patient-participants	As reported: *6 of the parents experienced RbG recruitment and 17 did not*.	*n* = 23		As reported, most of the participants were *mothers, Caucasian, and had at least a bachelor’s degree*.	US
[27]	IRB chairs' perspectives on genotype-driven research recruitment	2012	Qualitative research, Survey with commercial and institutional IRBs	Targeted institutional and commercial IRBs in the US	*n* = 201	50% completed the survey	As reported, most of the participants were *white, non-Hispanic males, age 50 or older. The most IRBs reported more than 4 years of service as an IRB chair and had a professional background in medicine or social science*. As reported, over 80% chose *“academic institution” as the best descriptor of their current institution and 17% IRBs reported to have been involved in reviewing a protocol involving genotype-driven recruitment*.	US (online survey)
[28]	Recommendations for ethical approaches to genotype-driven research recruitment	2012	Workshop, Multisite study, workshop with multiple stakeholders, including some study participants - discussion was informed by empirical data of [21] - in-depth interviews with research participants in six studies where genotype-driven recontact occurred [1–3, 22]	A wide range of stakeholders	*n* = 34 (affirmed their agreement on the final recommendations)		As reported, stakeholders in RbG research: *researchers, study coordinators, and participants from studies, as well as bioethics scholars, IRB leaders, other genomic and biobank researchers, clinicians, and federal officials engaged in issues related to human subjects research*.	US
[29]	Am I a control?: Genotype-driven research recruitment and self-understandings of study participants	2012	Qualitative research, same interviewees as for [2]	The eligibility criteria included the presence of one of two genetic variants. (*Cystic Fibrosis participants n = 9; Biobank “healthy volunteers” Participants n = 15*)	*n* = 24		As reported, all 24 interviewees were *self-reported as White and non-Hispanic (reflecting the racial and ethnic composition of the study population). The respondents were generally well educated, particularly the biobank participants*.	US
[7]	The ethics conundrum in Recall by Genotype (RbG) research: Perspectives from birth cohort participants	2018	qualitative research, semi-structured interviews	The purposive sampling strategy sampled participants across three categories: *(1) general ALSPAC cohort participants who had never participated in an RbG study;* *(2) participants who had participated in an ALSPAC RbG study; and* *(3) individuals who had served on one or more ALSPAC committees at which RbG study applications were discussed*	*n* = 53	The final response rate (26.5%) (*200 ALSPAC participants received an invitation email and 74 expressed interest)*.	As reported, the participants are *young adult participants of the Avon Longitudinal Study of Parents and Children (ALSPAC)*. As reported, of the *53 participants interviewed, 29 were female, and 51 has been enrolled in ALSPAC continuously since birth*.	UK

### Data with and without context

We identified significant differences between RbG studies accompanied by qualitative or empirical research and those without an empirical element. Furthermore, we found that empirical research on ELSI of RbG derives from US- or UK-based studies. We identified differences in how publications reported and contextualised participation rates of quantitative RbG studies and response rates for qualitative and empirical studies, as shown in Table 2. These differences need further attention. Some of the differences stem from and relate to the specifics of the respective RbG research study and its population and are therefore to be discussed in the context.

### ELSI aspects in different RbG study designs and policies

The targeted genotype-driven research approach tests a hypothesis. Accordingly, the researchers can better anticipate and communicate the potential research results for prospective or recalled participants than in untargeted WGS and GWAS [5, 21, 23, 33]. In line with other research, we identified a lack of consensus and standardised approaches, methods and boundaries to classify and communicate the clinical validity and utility of the individual carrier status from the original study (WGS or GWAS) [6, 23]. However, participants might not be informed about why they are eligible if the genetic results, which are the reason for eligibility, are not disclosed [32]. This, in turn, might invalidate the participant’s informed consent for the respective study. We identified a lack of a best practice on the decision to explicitly or implicitly obtain re-consent for RbG follow-up or substudies. We found no consensus on best practice on whether it is necessary to disclose the carrier status with uncertain clinical utility/validity. Likewise, we identified substantial differences in strategies to explain the study objective (in-depth or more general) and distinct reactions of participants and patients to the different disclosure strategies [6, 23].

We identified review studies foreseeing “*ethical barriers*” and concerns linked to the use of “*presumed*” consent where the explicit consent from older cohorts (or potentially dead participants) is not given due to cost and time [6]. In such studies, a waiver of consent from an ethical Review board enabled the RbG or another strategy where study policies were adjusted to an opt-out model [6, 32]. For example, an Icelandic genomics company successfully reasoned that explicit informed consent was unnecessary because of public support and security measures [6].

Publications on family-based recruitment in RbG reported that consent requirements should be left to the investigators and the IRB [21]. Some US-based IRBs preferred not to disclose individual results due to statements from the original consent and potential negative consequences. Studies demonstrated a high level of heterogeneity in IRB members’ views. However, most IRBs prioritised avoiding disclosure of genetic information with uncertain clinical utility rather than prioritising participant autonomy to make judgments and draw conclusions about the usefulness of the data [27]. There is a lack of standard practice on several key aspects: deciding whether it is necessary to explicitly or implicitly disclose the carrier status and design a suitable communication strategy in alignment.

### ELSI debates about disclosure by invitation and the “is there something wrong with me”? question

There have been recommendations to reduce distress for participants in the recall phase by involving the same elements as “trusted researchers” or the same institution as the original study and highlighting that an invitation to the specific RbG does not imply a particular genotype or phenotype [22, 25]. However, this explanation of the study design and eligibility criterion for the respective RbG study can evoke different reactions depending on the respective disclosure and communication strategy employed and the person’s individual experience. For example, some participants in a study investigating gene variants associated with epilepsy were confused by unsubstantiated findings in a study investigating gene variants associated with epilepsy, the disclosure of unsubstantiated findings confused some of the participants’ [23]. We identified substantial differences between the different groups as participants and patients. The reactions to the study invitation range from concerns as “*Why? Did you find something wrong with me?* [23]” to “*am I a control* [29]” assumptions about the individual’s group membership to perceptions on RbG study as “*just another study*” [7].

The heterogeneity of reactions stems, among other factors, from the fact that the participants feel different motivations depending on whether they are part of the “healthy” population group as controls or patients with a manifested genetic disease. Some individuals are carriers of a genetic variation that may or may not be disease-causing. Others are carriers of a genetic trait that does not (yet) have a corresponding phenotype. Whereas patients may experience no or low levels of concerns, healthy participants may assume or derive meaning when being invited to a RbG study [5, 19, 20, 24–26, 29]. There are significant differences in how the two groups conceptualised genetic research results as meaningful and accordingly also the preferences for receiving the results diversified [26]. However, the relevance of validity and/or utility linked to research results appeared in interviews with healthy participants and patients [26]. Some participants of these interviews carried an underlying “*bad news*” assumption in which results are implied to offer negative but definite information about a genetic condition, although the information material and invitation to the study stated otherwise [26]. These different reactions lead to concerns about the potential distress, uncertainties or anxieties triggered by the recall and disclosure strategy in the scientific community [23–25, 29].

Caution is needed to avoid cascade effects triggered by the assumed meaning because potentially harmful or unnecessary efforts to confirm findings with uncertain significance have been highlighted [24]. To avoid some of the mentioned distressed reactions to the recall process, a few studies decided not to disclose the targeted genetic variant and use more general language when describing the study objective [10, 23, 33–35]. In another strategy of an empirical study with RbG, participants reported about the use of a non-disclosure policy that was developed with a community board and accepted by participants because of: trust, a limited literacy on genetics and modest interest in research outcomes, and the perceived role in research participation as*”data providers* [7]”.

Qualitative data from interviews revealed that participants exhibit a high degree of heterogeneity in deciding whether to obtain research results with uncertain validity, but a consensus regarding the researcher’s “*duty to tell*” why they want to study their specific genetic sample [19]. The desire to know might negatively affect participation rates if participants are not provided with an explanation for why they are eligible for a specific RbG. It could perpetuate uncertainties and assumptions on clinical or personal utility or the reasons of eligibility for the RbG study. In cases where the investigated genetic variant is linked to a stigmatising condition, the decision to conceal or disclose the genetic variant “*cannot be made without the input of participants themselves”* [7]. We suggest further empirical research about participant engagement and involvement in study governance decisions to fine-map the considerations necessary for different study designs and informed consent procedures.

### ELSI debates and the quest for balance

We identified differences in how the recall and invitation process is designed and linked recommendations, from no disclosure—[10, 23, 33–35] to implicit disclosure [28, 34, 35] to explicit disclosure of the study objective or genetic variant targeted. Similarly, we identified differences in what kind of consent and RoRR policy was employed and discussed [6, 7, 10, 19–21, 25, 27, 28, 30, 31, 34]; from broad and lifelong to detailed, informed electronic and dynamic; as shown in Tables 1 and 2.

Many publications addressed the importance of balance and the implications on designs and policies for RbG, from balancing the scientific interests and the participant rights and preferences to balancing the protection of research participants while avoiding overly restrictive policies and the balance between the potential harms with or without disclosure. Some of these ELSI risks might be partially minimised by adding a sub-group of randomly or voluntarily selected participants to the specific RbG study sample group [25, 33]. Other publications reported on identified ELSI risks and harms of RbG in terms of a lack of representativeness and inclusivity. These risks associated with diversity and utility require more ELSI considerations and research and performative dimensions that tackle the lack of diversity and translational benefit and health disparities in genomics in general [12, 37].

## Discussion

Considering the urgency for shared ethical and legal frameworks to use the abundance of available genomic, geno- and phenotypic data, understanding and mapping the ELSI uncertainties is crucial to the evolution of RbG [8, 38]. ELSI challenges in RbG were thoroughly analysed in 2013 and raised the concern that this study design is not yet outlined with concise recommendations to use the approach in a broader spectrum of research [31]. However, are we at the point yet, where we have outlined and refined the study design appropriately to use it in a broader spectrum of research?

When designing invitation and disclosure strategies, there is no easy, one-size-fits-all solution to decide about whether, how and when to disclose the individual carrier status during the invitation process or not [23, 24, 39].

The analysis of the empirical studies confirmed the lack of qualitative data from diverse stakeholders and contexts. Accordingly, more diversified empirical studies about the context and outside the US/UK are needed. This research is crucial to understanding the differences in patients’ and healthy participants’ reactions to RbG study invitations.

The ethical and social/societal challenges presented by RbG need more empirical research to contextualise quantitative data as participation and response rates with qualitative data. Furthermore, this contextualisation of participation and response rates might help determine weak spots or a lack of understanding in the communication trajectory surrounding the respective RbG study. Measures to check the understanding of participants (of the study design, the information provided in the informed consent....) in RbG would therefore be valuable.

Similarly, the ELSI of disclosure of the individual carrier status with uncertain clinical utility might need more clarification in the communication trajectory with participants.

Acknowledging the contextual aspects of RbG through empirical, qualitative and normative research will refine the frameworks for RbG in the quest to find balances.

RbG studies can not be seen as isolated; the context and the consent of the original study shape the study design for the sub-study significantly. To find balance and to tailor the study policies to the context, the specific cases should be informed by anticipatory research on the specific RbG study and the context to the parental or original study. Providing tailored approaches that can cope with the identified heterogeneity of preferences and expectations of stakeholders requires tracing “*unruly ethics*” with qualitative research [40]. These empirical insights will provide a better understanding of the possibilities and limitations of upstream engagement of participants and patients to re-fine ELSI frameworks. Some of the identified ELSI issues in RbG are termed as “*ethical conundrums*” because they are novel challenges needing balances to respect the principle of autonomy adequately and not compromise the “*right not to know*” [7]. Many ethical dilemmas of RbG, which can be framed as unruly or conundrums, are not yet addressed extensively enough to formulate a best practice and to have a consensus on the ethical approval of a recruitment or disclosure strategy. Further qualitative research is needed to redefine appropriate approaches for different RbG studies and contexts to overcome the difficulty of informing participants thoroughly about the particular RbG study without creating anxiety. This is especially true for RbG studies where the genetic variant can be a stigmatising factor, or the re-invitation is unexpected [7, 16].

Nevertheless, we identified a broad agreement that participants want to understand why they are eligible [7, 24, 29]. Given that actionability and personal utility are drivers of participation, more research is needed on expectations in RbG and respective RoRR policies and linked communication to participants. The development and advancement of efficient electronic communication tools that decrease the time for recruitment and consent procedures require further attention to avoid overburdening participants and families, violating public trust or implicit social contracts and affecting the willingness to participate negatively [22, 31, 41].

However, obtaining viable sampling frames for specific RbG research studies will remain a practical, financial and logistic challenge. Utilising other population data may be a strategy to obtain statistical power, quality-control and produce robust science (to avoid mismatches as in the example of a Greenland-and Japan-based study of associations between variants in Type 2 Diabetes [4, 8]). Nevertheless, there are legal and ethical challenges with big data and cross-border sharing for global research approaches because legal compliance alone does not address the safeguarding function necessary for the complexity of data-driven research [38, 42, 43]. RbG studies require further ELSI considerations to safeguard research participants, collectively and individually, from potential unexpected discrimination to provide more than the legal and ethical minimum through technical measures such as pseudonymisation [38]. In research settings in low-income countries, the increasing involvement of commercial interests and industries, paired with weak governance structures and the unlikely immediate translational benefit from the commercialisation of genomics, may decrease trust and the willingness to participate in genomic research [13, 44]. Further societal considerations on how to increase diversity and inclusivity for GWAS, WGS and RbG sub-studies are needed to tackle the unequal access to translational benefits from and in genomic research and precision medicine [12, 45, 46].

We identified clear points of contention between researchers and research ethics review committees. These stem from utilising substantial distinct ethical frameworks that guide decisions regarding socioeconomic and practical factors [47, 48]. Some researchers discouraged the sharing of RbG research results with uncertain validity because of, among other things, not enough time and economic resources to implement processes to prevent these issues [24, 49]. We identified various prioritising schemes in defining the necessity to obtain re-consent for various RbG studies. This was due to the acknowledgement of different threats from the more comprehensive data-sharing environment to justify not obtaining consent, as in the cases of the “*care.data scheme* [50]” and the “*Icelandic case* [6]”. Presumed accordance with changes towards an opt-out consent model and life-long static consents and institutional and legal solutions that enable the re-use of data for RbG might run into the risk of violating trust because of being the ethical minimum [6, 50]. The focus on institutionalised bioethics and individual consent for data-driven research settings are insufficient to address ethical and social/societal challenges [16, 39, 47, 51].

Other RbG pilot studies included concise premises as eligibility requirements in the original consent procedure about the recall, communication and return of genetic findings to allow recalling participants of the original study for further RbG with an ethically sound strategy [16]. Approaches, with a dynamic consent and specified choices in terms of recall (for which studies) and if and how research results (with un or certain clinical utility) can enter the real-time of the individual, may address some ELSI concerns of RbG. By this, participants’ can adapt the given consent to their changing perspectives and needs, which is not given in the case of a life-long consent. Even if dynamic consent may be a partial solution to some shortcomings in consent procedures, more empirical cases are needed to determine how to provide the highest utility of dynamic consent models [52].

However, these apparent differences in strategies to conduct RbG studies lead to concerns about scientists’ self-governance. Research practices should be transformed by ethics and not be limited to adapting the consent models and using ethics as a ritualistic language to “*smooth over moments of dislocation*” and the political dimensions of practices [53]. Because of endemic problems such as the differences in knowledge, epistemological biases and pressing financial and time issues, making “*the ELSI perspective heard* [54]” does not suffice to have a real-time ELSI influence on policies in genomics.

In conclusion, this review led to an overview of ELSI in RbG and shed light on understudied issues that require further qualitative research. The findings herein serve to map and generate an understanding of the different stakeholder’s perspectives on the ELSI strategies used in RbG studies that need to be investigated further. We identified areas with compelling or contrasting qualitative research results that require further attention and clarification to re-fine ELSI frameworks for RbG. These findings contribute to the further development of qualitative studies linked to RbG follow-up or sub-studies in large research and biobanking repositories.

## Supplementary information

Appendix
