# Supplementary material for: Ethical, legal and social/societal implications (ELSI) of recall-by-genotype (RbG) and genotype-driven-research (GDR) approaches: a scoping review
Source: Eur J Hum Genet. 2022 Jun 15;30(9):1000–10. doi: 10.1038/s41431-022-01120-y (PMC9437022; doi:10.1038/s41431-022-01120-y)
Supplement: Supplementary file 1 — Appendix [file 41431_2022_1120_MOESM1_ESM.docx]

## Appendix

Detailed list of the included publications and the thematic and content analysis of the publications

| Year | Ref | title | type of document | journal | method & target sample | ethic*, legal*, social* or societal* terms found |
| --- | --- | --- | --- | --- | --- | --- |
| 2004 | (19) | Ethical issues in identifying and recruiting participants for familial genetic research | Research Review | Am J Med Genet A | Literature review | - ethical issues, ethical concerns, ethical considerations, ethical principles, ethical barriers, ethical and scientific implications, ethically responsible research, ethically acceptable, ethical ramifications, ethical reason  - societal benefits, societal value, social science research |
| 2008 | (20) | Don't throw the baby out with the bathwater: enabling a bottom-up approach in genome-wide association studies | Editorial Material | Genome Res | Forum | - ethical considerations, centralized ethics review board |
| 2010 | (21) | Ethical challenges in genotype-driven research recruitment | Commentary | Genome Res | Case presentation | - ethical challenges, ethics consultation, research ethics consultation, basic ethical principles underlying the consent language, ethically appropriate disclosure, advice, and referral, ethical issues,  ethical considerations of respect for persons, beneficence, paternalism, reciprocity, and the boundaries between research and clinical practice |
| 2010 | (22) | Personal genome research : what should the participant be told? | Editorial Material | Trends Genet | Current practice and policy | - ethical challenges associated with whole genome sequencing (WGS) research is what to communicate to study participants?, ethical ‘imperative’ to return results in genetic research, ethical practical and scientific considerations, ethical commitment in research,  - no societal but social science studies  - legal guidance, moral or legal obligation to return results of unproven significance |
| 2011 | (23) | Research participants' perspectives on genotype-driven research recruitment | Journal Article | J Empir Res Hum Res Ethics | qualitative research, interviews with participants | - ethical challenges, ethically responsible research, ethically responsible research, interviewees’ perspectives on ethical and policy issues, ethical issue,  - ethical challenges stemming from the use and possible disclosure of genetic research results as part of the offer to participate in additional research |
| 2011 | (24) | The meaning of genetic research results: reflections from individuals with and without a known genetic disorder | Journal Article | J Empir Res Hum Res Ethics | qualitative research, in-depth interviews | - researchers and ethics boards,  - ethical, legal, and social implications |
| 2011 | (25) | Epilepsy patient-participants and genetic research results as "answers" | Journal Article | J Empir Res Hum Res Ethics | qualitative research, semi-structured interviews | - ethical challenges, ethical dilemmas surrounding the complex and muchdebated issue of return of individual genetic research results |
| 2011 | (26) | Parent perspectives on pediatric genetic research and implications for genotype-driven research recruitment | Journal Article | J Empir Res Hum Res Ethics | qualitative research, interviews with parents of epilepsy patient-participants | - ethical considerations about the use and disclosure of genetic information as part of the recruitment process, ethical challenges in pediatric genetic research studies that intersect with ethical issues involving genotype-driven research recruitment |
| 2012 | (27) | IRB chairs' perspectives on genotype-driven research recruitment | Journal Article | IRB Ethics and Human Research | Qualitative research, Survey with commercial and institutional IRBs | - ethical challenges, ethically acceptable approaches to genotype-driven recruitment depending on context,  - ethical Dilemmas: Weighing the Issues, (ethical concerns shifted to the recruitment phase when genetic information that is generated in one study is used as the basis for identifying and recontacting participants about further research) |
| 2012 | (28) | Recommendations for ethical approaches to genotype-driven research recruitment | Journal Article | Human genetics | Workshop with wide range of stakeholders | - ethical challenges, ethical issues associated with recontacting participants for the purpose of additional research recruitment and with genotype-driven recruitment |
| 2012 | (29) | Am I a control?: Genotype-driven research recruitment and self-understandings of study participants. | Original research article | Genetics in medicine : official journal of the American College of Medical Genetics | Qualitative research, Multisite study, workshop with multiple stakeholders | - recommendations for ethical approaches to genotype-driven research recruitment |
| 2012 | (30) | Balancing the risks and benefits of genomic data sharing: genome research participants' perspectives | Original paper | Public Health Genomics | randomized trial of three consent types, follow-up interviews | - advance the ethical conduct of genome research, general societal benefits of participating in research  - ELSI |
| 2013 | (31) | Genotype-driven recruitment: a strategy whose time has come? | Journal Article | BMC Med Genomics | Debate | - ethical issues, ethical concerns  - societal implications of recruiting individuals in original GDR studies  - questions related to the legal authority of these committees may emerge, legal framework |
| 2014 | (32) | Biobanks and personalized medicine | Review | Clin Genet, | Mini-review | ethical concerns |
| 2016 | (4) | Statistical power considerations in genotype-based recall randomized controlled trials | Journal Article | Scientific reports | Article, Comparative study | specific ethical considerations for GBR trials |
| 2017 | (33) | Genotype-Driven Recruitment and the Disclosure of Individual Research Results | Editorial Material | Am J Bioeth | Editorial Material | - several ethically acceptable approaches to genotype-driven recruitment depending on context  - plans for minimizing risks and managing ethically appropriate disclosure, advice, and referral |
| 2017 | (34) | Harms of Deception in FMR1 Premutation Genotype-Driven Recruitment | Editorial Material | Am J Bioeth | Editorial Material | - ethical questions about the nature of the study  - ethical duties to disclose results |
| 2017 | (35) | Genotype-Driven Recruitment Without Deception | Editorial Material | Am J Bioeth | Case commentaries | - ethical challenges for genotype-driven research |
| 2017 | (10) | Genotype-Driven Recruitment in Population-Based Biomedical Research | Case report | Am J Bioeth | Case report | - ethical and research design reasons  - ethical concerns regarding the disclosure  of research results in the context of genotype-driven recruitment  - ethical consent process |
| 2017 | (36) | Genetics of coronary artery disease: discovery, biology and clinical translation | Review | Nature Reviews Genetics | Review | - alteration of an individual’s DNA raises a host of ethical and social questions |
| 2018 | (5) | Formalising recall by genotype as an efficient approach to detailed phenotyping and causal inference | Review | Nat Commun | Review and comparative study | - ethical challenges associated with recruitment by genotype  - specific ethical issues  - ethical principles of respect and reciprocity  - ethical balance for RbG studies |
| 2018 | (6) | Genotype-Based Recall Studies in Complex Cardiometabolic Traits | Review | Circ Genom Precis Med | Review | - ethical barriers |
| 2018 | (7) | The ethics conundrum in Recall by Genotype (RbG) research: Perspectives from birth cohort participants | Research Article | PloS one | qualitative research, semi-structured interviews with participants | novel ethical context and challenges, ethical principle of autonomy, basis of informed consent in contemporary research is pitched against the ‘right not to know’, non-maleficence (the precept to do no harm)  - participants demonstrated societal solidarity  - ethical, legal or other normative principles |
| 2020 | (8) | Unique roles of rare variants in the genetics of complex diseases in humans | Review | Journal of Human Genetics | Review | - ethical, social, and legal implications of population screenings conducted mainly in specific high-risk populations, |
| 2020 | (9) | Cohort Profile: East London Genes & Health (ELGH), a community-based population genomics and health study in British Bangladeshi and British Pakistani people | Article | Int J Epidemiol | Cohort profile | - subject to ethics approval  - societal: overrepresentation of specific populations and underrepresentation of others |
